# Bioconversion of duck blood cell: process optimization of hydrolytic conditions and peptide hydrolysate characterization

**DOI:** 10.1186/s12896-018-0475-5

**Published:** 2018-10-20

**Authors:** Zhaojun Zheng, Xubiao Wei, Tingting Shang, Yan Huang, Cong Hu, Rijun Zhang

**Affiliations:** 1 State Key Laboratory of Animal Nutrition, College of Animal Science and Technology, China Agricultural University, Beijing, 100193 China; 2 Department of Computer Science, College of Information and Electrical Engineering, China Agricultural University, Beijing, 100083 China

**Keywords:** Antioxidant activity, Degree of hydrolysis, Functional properties, Peptide hydrolysate, Response surface methodology

## Abstract

**Background:**

As the protein-laden by-product, red blood cells (RBCs) from poultry blood is a potential source of protein used as food and feed ingredient. However, RBC was currently underutilized. Therefore, it is an urgent need to develop feasible and cost-effective methods for converting poultry waste into nutritional and functional products.

**Results:**

To take full advantage of this poultry waste, peptide hydrolysate was produced by deep controllable bioconversion of RBC, by means of synergistic combination of neutrase and flavourzyme. In this work, the functional properties and antioxidant activity of peptide hydrolysate were also characterized. The degree of hydrolysis (DH) was optimized using response surface methodology, and optimal hydrolysis conditions were found to be: temperature 51 °C, substrate concentration 14% (*w*/*v*), initial pH 7.0, and time 7.5 h. The red blood cell hydrolysate (RBCH) obtained not only possessed plentiful small peptides (< 3 kDa, 68.14%), but also was abundant in essential amino acids, accounting for over 50% of total amino acids. In addition to its excellent solubility (> 80%), emulsifying and foaming properties, RBCH also exhibited notable antioxidant activities, such as DPPH (2,2-diphenyl− 1-picrylhydrazyl) radical-scavenging activity (IC_50_, 4.16 mg/mL), reducing power, metal chelating ability and inhibiting lipid peroxidation.

**Conclusions:**

RBCH enriched in small peptides has the potential to be a new food additive with outstanding functional and antioxidant properties, and a process was established for converting poultry waste into peptide hydrolysate using neutrase and flavourzyme.

**Electronic supplementary material:**

The online version of this article (10.1186/s12896-018-0475-5) contains supplementary material, which is available to authorized users.

## Background

In recent years, recycling of wastes from agricultural and food processing has been regarded as a significant industrial procedure to alleviate the shortage of protein resource. Animal blood derived from slaughterhouse waste is a potential edible protein source for human consumption [1]. Red blood cell (RBC), the major fraction of blood, accounts for about 60% of the total protein content, which is currently underutilized and discarded [2]. Therefore, finding ways to utilize RBC is necessary for maximizing the economical benefits and reducing the problems associated with its disposal.

Enzymatic hydrolysis is a particularly attractive technique to convert underutilized RBC into the acceptable and marketable forms. Intriguingly, enzymatic hydrolysis offers several advantages, including mild reaction conditions, high product yields, minimal formation of by-products, high safety and low energy cost [3]. Proteolysis is measured by degree of hydrolysis (DH), which is a key parameter to determine the nutritional and functional properties of protein hydrolysate [4, 5]. Thus, it is important for optimizing DH and its involved parameters, such as enzyme concentration, substrate concentration, reaction temperature, hydrolysis time, and pH, to achieve an economical and nutritional protein hydrolysate. Response surface methodology (RSM) is an effective technique applied to optimize food processing operations, which can employ several variables simultaneously and describe the overall process [6, 7].

In addition to its fundamental nutritional role, the bio-functional features of RBC are now being recognized [8]. It has been shown that protein hydrolysates possess several desirable functional properties, which can be modified by the elevated soluble nitrogenous compounds resulted from the enzymatic hydrolysis [9]. As DH increases the solubility of hydrolysate increases, and it further affects other functional properties like foaming and emulsification [10, 11]. Furthermore, high solubility of protein hydrolysate is a substantially beneficial feature for many food applications. Apart from the functional properties, protein hydrolysates from animal protein sources such as fish [12] and porcine plasma [13], have been shown to possess antioxidant activity to eliminate free radicals and inhibit lipid peroxidation. Moreover, protein hydrolysate with antioxidant activity is a potential antioxidant alternative for its higher concentration without the deleterious effects associated with the use of synthetic equivalents.

To the best of our knowledge, RBCs that are used as sources for preparing hydrolysates are generally obtained from porcine and bovine sources, while the utilization of poultry blood is limited. In this work, we first studied the synergetic effect of neutrase and flavourzyme on the protein derived from duck blood. The processing technology for enzymatic hydrolysis of duck blood cell was subsequently optimized by means of RSM. Importantly, the functional and antioxidant properties of protein hydrolysate as well as its nutritional value were investigated.

## Methods

### Chemicals and reagents

Neutrase (actual activity of 40,000 U/g) and flavourzyme (actual activity of 10,000 U/g) for protein hydrolysis were purchased from Nanning Pangbo Biological Engineering Co. Ltd. (Nanning, China). 2,2-diphenyl− 1-picrylhydrazyl (DPPH), ferrozine, linoleic acid, butylated hydroxyltoluene (BHT), ascorbic acid and tocopherol were obtained from Sigma-Aldrich (St. Louis, MO, USA). All other chemicals used were of analytical reagent grade and obtained from commercial sources.

### Sample preparation

Duck blood in this study was sampled from Yangyuan Biological Engineering Co., Beijing, China. Fresh duck blood was immediately centrifuged at 8000×*g* and 4 °C for 10 min in a High Speed Refrigerated Centrifuge to separate plasma (supernatant) and red blood cell (bottom layer). The red blood cell was kept frozen at − 20 °C until use.

### Central composite design

Based on the manufacturer’s information and preliminary experiments of enzymatic hydrolysis, four controllable variables (temperature, substrate concentration, initial pH, and time) were chosen for optimization by RSM using a Central Composite Design (CCD) of experiments (Table 1). Upon the completion of experiments, the mathematical relationship between response (DH) and variables (temperature, substrate concentration, initial pH, and time) was described with a quadratic model as follow:

1 $$ \mathrm{Y}={\mathrm{a}}_0+{\mathrm{a}}_1{x}_1+{\mathrm{a}}_2{x}_2+{\mathrm{a}}_3{x}_3+{\mathrm{a}}_4{x}_4+{\mathrm{a}}_{12}{x}_1{x}_2+{\mathrm{a}}_{13}{x}_1{x}_3+{\mathrm{a}}_{14}{x}_1{x}_4+{\mathrm{a}}_{23}{x}_2{x}_3+{\mathrm{a}}_{24}{x}_2{x}_4+{\mathrm{a}}_{34}{x}_3{x}_4+{\mathrm{a}}_{11}{x}_1^2+{\mathrm{a}}_{22}{x}_2^2+{\mathrm{a}}_{33}{x}_3^2+{\mathrm{a}}_{44}{x}_4^2 $$

Where Y is a predicted response, a_0_ is a constant coefficient (intercept), a_1_, a_2_, a_3_ and a_4_ are linear effects, a_12_, a_13_, a_14_, a_23_, a_24_, and a_34_ are interaction effects.

**Table 1:** Central composite design matrix, experimental and predicted values for response surface analysis

Run	Independent variables								Response	
	Coded value				Actual value				DH (%)	
	*x*_1_	*x*_2_	*x*_3_	*x*_4_	T^1^ (°C)	C^2^ (%)	pH	Time (h)	Observed	Predicted
1	−1	1	1	−1	45	18	8.0	6.0	25.45	25.42
2	−1	1	1	1	45	18	8.0	7.0	26.46	26.99
3	1	1	−1	−1	55	18	7.0	6.0	28.77	28.58
4	2	0	0	0	60	16	7.5	6.5	23.64	24.14
5	0	2	0	0	50	20	7.5	6.5	30.66	30.77
6	0	0	0	0	50	16	7.5	6.5	31.26	31.91
7	0	0	0	0	50	16	7.5	6.5	31.46	31.91
8	1	−1	−1	−1	55	14	7.0	6.0	28.14	27.73
9	1	-1	-1	1	55	14	7.0	7.0	30.55	30.83
10	1	-1	1	1	55	14	8.0	7.0	31.68	31.54
11	0	0	0	0	50	16	7.5	6.5	32.16	31.91
12	−2	0	0	0	40	16	7.5	6.5	19.13	18.27
13	−1	−1	1	−1	45	14	8.0	6.0	25.82	26.15
14	1	1	−1	1	55	18	7.0	7.0	31.17	30.96
15	0	0	0	0	50	16	7.5	6.5	31.81	31.91
16	0	0	0	0	50	16	7.5	6.5	32.56	31.91
17	1	−1	1	−1	55	14	8.0	6.0	28.30	28.56
18	−1	1	−1	−1	45	18	7.0	6.0	25.54	25.80
19	−1	1	−1	1	45	18	7.0	7.0	27.50	27.49
20	0	0	−2	0	50	16	6.5	6.5	29.79	29.83
21	0	0	0	0	50	16	7.5	6.5	32.38	31.91
22	1	1	1	−1	55	18	8.0	6.0	28.97	28.86
23	0	0	0	2	50	16	7.5	7.5	33.61	33.27
24	−1	−1	−1	1	45	14	7.0	7.0	28.15	28.38
25	0	0	2	0	50	16	8.5	6.5	30.57	30.16
26	0	−2	0	0	50	12	7.5	6.5	31.84	31.36
27	0	0	0	−2	50	16	7.5	5.5	28.63	28.60
28	1	1	1	1	55	18	8.0	7.0	31.21	31.12
29	−1	−1	1	1	45	14	8.0	7.0	28.00	28.44
30	−1	−1	−1	−1	45	14	7.0	6.0	25.64	25.98

^1^Temperature

^2^substrate concentration

### Enzymatic hydrolysis

The peptide hydrolysate was prepared according to the previous method [14]. Briefly, RBC was adjusted to 7.0 using 1.0 M NaOH or lactic acid (85%) and neutrase (3000 U/g) and flavourzyme (1000 U/g) alone or together were added to the solution. The mixture was incubated at 50 °C and 200 rpm, and 5 mL sample was collected every 60 min to detect the DH. Later, the optimization of enzymatic hydrolysis was carried out according to the experimental design described in Table 1. The peptide hydrolysate was dehydrated by spray drying in a laboratory spray dryer (SD-Basic Spray Dryer, LabPlant, England) with an inlet temperature of 130 °C, outlet temperature of 80 °C.

### Determination of the degree of hydrolysis

The DH was determined with the o-phthaldialdehyde method described by PM Nielsen, D Petersen and C Dambmann [15]. 400 μL sample was mixed with 3 mL OPA reagent, and incubated at room temperature for exactly 2.0 min. Then the absorbance of the mixture was measured at 340 nm in a UV-visible spectrophotometer (Pgeneral, Beijing, China).

### Determination of molecular weight distribution

The molecular weight distribution profile of the sample was estimated by high-performance gel-filtration chromatography using ÄKTA avant 25 chromatography system and Superdex 75 10/300 GL column (GE Healthcare, Uppsala, Sweden). Samples (10 mg) were dissolved in 1 mL of 0.01 M Tris-HCl solution and centrifuged at 12,000 rpm for 10 min. Then supernatant was injected for analysis by HPLC. The results were obtained using UV detector, and the molecular weight was calculated using a calibration curve produced by various known concentrations of standard proteins.

### Amino acid composition

Amino acid compositions of protein and hydrolysate were measured by the amino acid auto-analyzer (Hitachi High-Technologies Corporation, Japan). Fifteen of amino acids were determined after 24 h of acid hydrolysis using 6 M HCl at 110 °C. Methionine and cystine were determined from 24 h acid hydrolysates following formic acid oxidation of the samples. The amount of each amino acid in the samples was calculated and expressed as a percentage of the total sample weight.

### Determination of functional properties

#### Solubility

Solubility was determined in triplicate according to the method of I Suppavorasatit, EG De Mejia and KR Cadwallader [16] with some modifications. 200 mg of samples were dispersed in 20 mM citrate-phosphate buffers of various pH values. All samples were stirred at 25 °C overnight and then centrifuged at 5000×*g* for 15 min. Protein in the supernatant and total protein in sample were determined by the BCA Quantitation Kit (Beijing BLKW Biotechnology Co., Ltd.). The solubility was calculated as follows:

2 $$ \mathrm{Solubility}\ \left(\%\right)=\left[\left(\mathrm{protein}\ \mathrm{in}\ \mathrm{supernatant}\right)/\left(\mathrm{total}\ \mathrm{protein}\right)\right]\times 100 $$

#### Emulsifying properties

Emulsifying activity index (EAI) and emulsion stability index (ESI) of protein samples were determined following the method of Q Liu, B Kong, YL Xiong and X Xia [13]. Emulsions of protein dispersions were prepared by mixing 10 mL of vegetable oil with 30 mL of 0.2% (*w*/*v*) protein dispersion in 20 mM citrate-phosphate buffer solution at different pH values. Then the mixtures were homogenized at 20,000 rpm for 1 min using a homogenizer (IKA T18, Staufen, Germany). An aliquot of the emulsion (50 μL) was immediately mixed with 5 mL of 0.1% (w/v) sodium dodecyl sulphate (SDS) solution for 15 min. The absorbance of mixture was measured at 500 nm with a UV-visible spectrophotometer. EAI and ESI were calculated as follows:

3 $$ \mathrm{EAI}\ \left({\mathrm{m}}^2/\mathrm{g}\right)=\left(2\times 2.303\times {\mathrm{A}}_0\right)/\left[0.25\times \mathrm{protein}\ \mathrm{weight}\left(\mathrm{g}\right)\right] $$

4 $$ \mathrm{ESI}\ \left(\min \right)=\left[{\mathrm{A}}_0/\left({\mathrm{A}}_0-{\mathrm{A}}_{15}\right)\right]\times 15 $$

where A _0_ and A _15_ are the absorbance at time 0 and 15 min.

#### Foaming properties

Foaming capacity and foaming stability of samples were evaluated according to the method described by A Kato, T Fugishing, N Matsudomi and K Kobashi [17] with some modifications. Protein dispersions (0.5%, *w*/*v*) prepared in 20 mM citrate-phosphate buffer at different pH values, were homogenized at 18000 rpm for 1 min using a homogenizer (IKA T18, Staufen, Germany). Foaming capacity was calculated as the percentage of increasing volume upon mixing. Foaming stability was expressed as the percentage of remaining foam after 60 min without disturbing.

### Evaluation of antioxidant activity

#### DPPH radical scavenging activity

DPPH radical-scavenging activity was determined according to the method of Sun et al. [18] with slight modification. 1.5 mL of sample solution was mixed with 1.5 mL of DPPH solution (1.0 mM in 95% ethanol) at room temperature for 30 min in the dark. The absorbance of reaction solution was measured at 517 nm. DPPH radical-scavenging activity was calculated according to the following equation:

$$ \mathrm{Scavengingactivity}\left(\%\right)=\left(1-\left({\mathrm{A}}_{\mathrm{sample}}-{\mathrm{A}}_{\mathrm{blank}}\right)/{\mathrm{A}}_{\mathrm{control}}\right)\times 100 $$

where A_sample_ was sample absorbance; A_blank_ was the blank absorbance without DPPH solution; A_control_ was the absorbance of control group without sample.

#### Reducing power

The reducing power was determined according to the method of A Taheri, KH Sabeena Farvin, C Jacobsen and CP Baron [19]. 1 mL sample was mixed with 1 mL of 0.2 M phosphate buffer (pH 6.6) and 1 mL of 1% (*w*/*v*) potassium ferricyanide. The mixture was incubated at 50 °C for 20 min and 1 mL 10% (w/v) trichloroacetic acid was added to this mixture. An aliquot of 2 mL from this incubation mixture was mixed with 2 mL of distill water and 0.4 mL of 0.1% (w/v) ferric chloride. After reaction for 10 min, the absorbance of reaction solution was measured at 700 nm. The reducing power is represented as the absorbance, and a higher absorbance indicates a stronger reducing power.

#### Metal chelating activity

The chelating activity on Fe^2+^ was determined according to the reported method A Taheri, KH Sabeena Farvin, C Jacobsen and CP Baron [19]. 1.0 mL of 20 μM FeCl_2_ was mixed with 1.0 mL of 0.5 mM ferrozine. The mixture was then reacted with 0.5 mL of sample for 20 min at room temperature. The absorbance was read at 562 nm. The ability to chelate the ferrous ion was calculated as follows:

$$ \mathrm{Chelatingability}\left(\%\right)=\left(1-{\mathrm{absorbance}}_{\mathrm{sample}}/{\mathrm{absorbance}}_{\mathrm{blank}}\right)\times 100 $$

where A_sample_ was the sample absorbance; A_blank_ was the blank absorbance.

#### Lipid peroxidation assay

The lipid peroxidation inhibition ability of sample was detected in a linoleic acid model system [20]. Briefly, the sample (5.0 mg) was dissolved in 10 mL of 50 mM phosphate buffer (pH 7.0) and added to a solution of 130 μL of linoleic acid in 10 mL of 99.5% ethanol. Then the total volume was adjusted to 25 mL with distill water. The mixture was incubated at 40 °C in a dark room, and the degree of oxidation was evaluated for 8 days by measuring ferric thiocyanate values. The reaction solution (100 μL) incubated in the linoleic acid model system was mixed with 4.7 mL of 75% ethanol, 0.1 mL of 30% (*w*/*v*) ammonium thiocyanate, and 0.1 mL of 20 mM ferrous chloride solution in 3.5% HCl. After 3 min, the degree of color development that represents linoleic acid oxidation was measured at 500 nm. The absorbance value indicates the degree of lipid peroxidation.

### Statistical analysis

ANOVA was used to determine the differences among treatments (*P* < 0.05) by the statistical program JMP 12.0 software package (SAS Institute Inc.) and Minitab 16 statistical software (State College, PA). The resulting data were investigated graphically using Origin 9.0 software (OriginLab, Northampton, MA).

## Results and discussion

### Synergistic effect of neutrase and flavourzyme

**Fig. 1:**
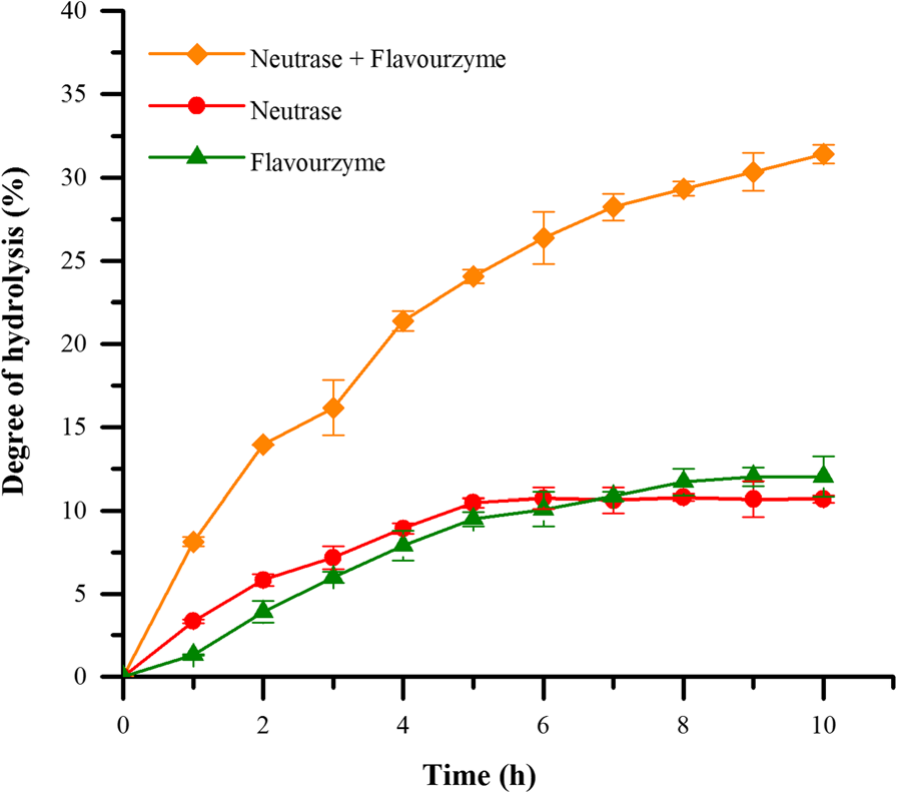
Time-course hydrolysis of protein from duck blood red cell. Separate hydrolysis: 3000 U/g neutrase (circle) or 1000 U/g flavourzyme (triangle); simultaneous hydrolysis: 3000 U/g neutrase and 1000 U/g flavourzyme (diamond)

To obtain a hydrolysate with high nutritional value, the protein hydrolysates should enrich low molecular weight peptides, directly correlating with the degree of proteolysis. Thus, neutrase and flavourzyme were incubated together or individually with RBC to evaluate the degree of enzymatic hydrolysis. Not surprising, DH increased over time until reach a plateau in the treatment of neutrase, flavourzyme or their combination (Fig. 1). Compared to the group with only neutrase or flavourzyme, DH was significantly higher when both two proteases were added. After 10 h incubation with both neutrase and flavourzyme, DH of hydrolysate reached 31.41 ± 0.56%. Comparably, DH reached 10.69 ± 0.21% and 12.02 ± 1.22%, respectively, for only neutrase or flavourzyme group. It was obvious that the mixture of neutrase and flavourzyme could cause higher hydrolysis of RBC as compared to the single protease treatment. Based on the results obtained above, we reasoned that endoproteinase (neutrase) combining with exopeptidase (flavourzyme) could accelerate the enzymatic hydrolysis and obtain great degree of degradation.

### Optimization of process parameters

Experimental data from CCD were obtained using 30 combinations of four factors: temperature, substrate concentration, initial pH, and time (Table 1). In terms of actual factors, an empirical relationship between DH and variables without considering non-significant terms could be expressed by the second-order polynomial equation below:

5 $$ {\displaystyle \begin{array}{l}\mathrm{DH}\left(\%\right)=-395.02+2.53{x}_1+9.64{x}_2+27.74{x}_3+14.34{x}_4+0.03{x}_1{x}_2\\ {}-0.14{x}_1{x}_3-0.18{x}_1{x}_4+0.07{x}_2{x}_3+0.07{x}_2{x}_4-0.05{x}_1^2-0.11{x}_2^2-1.91{x}_3^2-0.97{x}_4^2\end{array}} $$

According to the results of ANOVA for examining the model shown in Additional file 1: Table S1, F-value of 469.47 implied that the model was significant with a highly satisfactory value of *R*^*2*^ of 96.31%, which indicated that only 3.69% of the total variation was not explained by the model. The model also resulted in high value of the adjusted determination coefficient (adjusted *R*^*2*^ = 0.9610) with no significant lack of fit (*p* = 0.467 > 0.05), indicating the regression modeling procedure was acceptable.

**Fig. 2:**
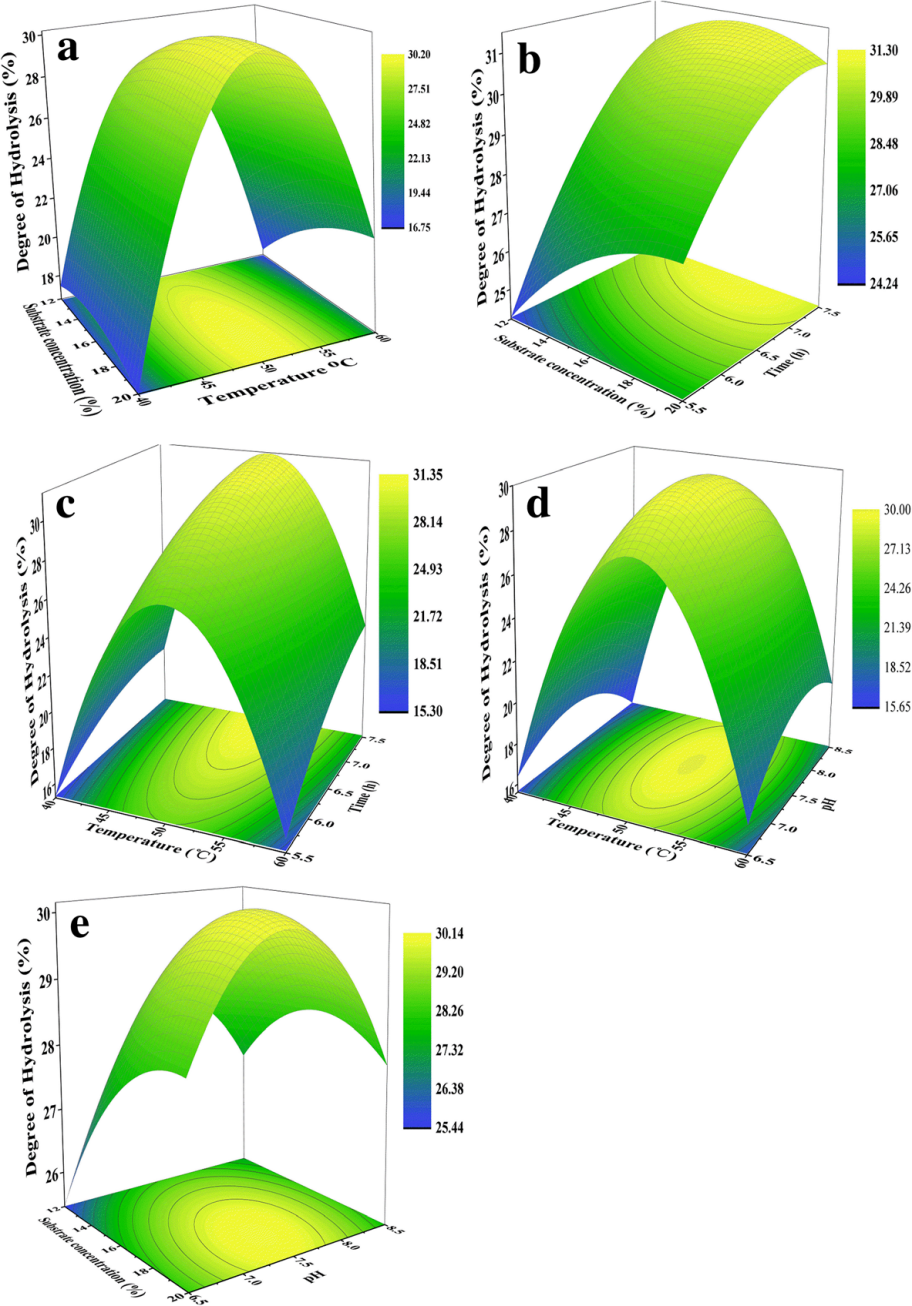
Response surface plots for degree of hydrolysis (DH) as a function of **a** temperature and substrate concentration, **b** substrate concentration and time, **c** temperature and time, **d** temperature and initial pH, **e** substrate concentration and initial pH

The second-order response surface plots were drawn to illustrate the main and interactive effects of the independent variables on the DH (Fig. 2). Temperature played a maximal linear influence on the DH, and this factor also had significant interactions with other three variables. DH increased as a function of temperature until around 49-51 °C, after which it dropped with further increases in temperature (Fig. 2a). Though not readily apparent, Fig. 2a showed that at lower temperature DH decreased with increasing substrate concentration; however, the lower-left corner of the response surface showed that at higher temperature DH increased with the rise of concentration. In contrast, the response surface in Fig. 2b showed that DH increased slowly with the rise of substrate concentration at shorter time, and DH declined with increasing substrate concentration over time, which was similar to the report of D Peričin, L Radulović-Popović, Ž Vaštag, S Mađarev-Popović and S Trivić [21] about the effects of time and enzyme concentration. This phenomenon occurred might be related to the inherent characters of the enzyme system whatever the substrate was. As the most important linear variables of influencing the DH values, temperature and time had a clear interaction effect on DH as shown in Fig. 2c. Unlike the correlation between DH and temperature, the DH values increased with increasing time, which was similar to the reports by other researchers [21–23]. Fig. 2d showed that initial pH played a minor role in the improvement of DH, but the interaction between pH and temperature was significant. Greater DH was obtained at high pH values, with the highest DH occurring at around pH 7.3–7.6, after which DH declined. This phenomenon could be explained by a loss of enzyme stability with the increment of pH [16]. As shown in Fig. 2e, the lowest DH was observed both at the combination of low substrate concentration and low pH or at high substrate concentration and high pH.

The numerical optimization of hydrolysis parameters was carried out using JMP Pro 12 software based on the initial experimental results. To obtain the highest DH, the experiment was conducted according to the predicted and optimal conditions of 51 °C temperature, 14% substrate concentration (*w*/*v*), 7.0 pH, and 7.5 h time. The result from confirmation experiment at optimum point was 32.06 ± 0.59% for DH that was predicted by the model as 32.80%. The experimental value agreed with the value predicted by the model within a 95% confidence interval, showing the validity of this response model.

### Molecular weight distribution

**Fig. 3:**
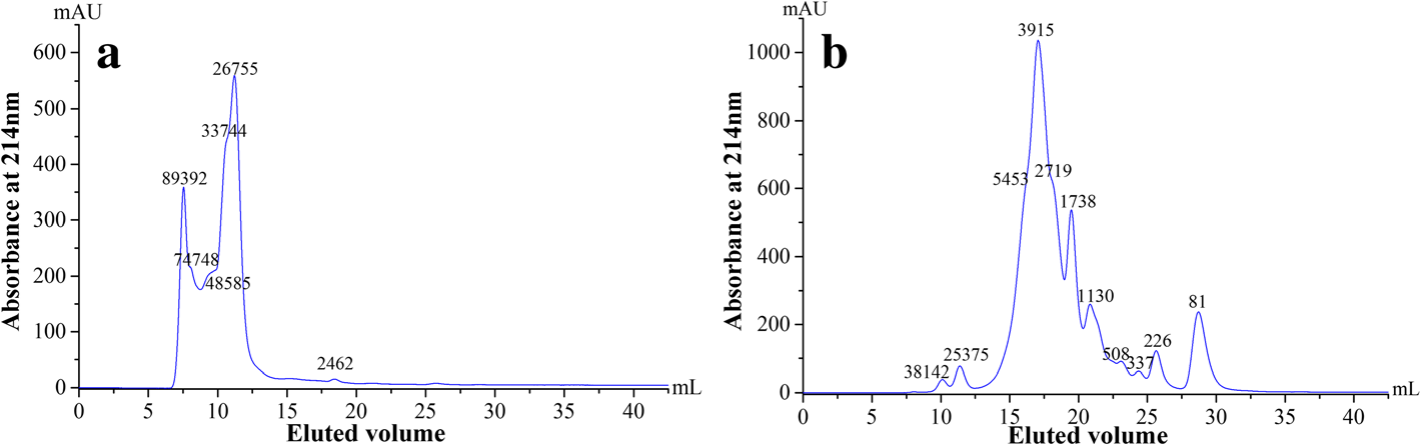
Molecular weight distribution of red blood cell protein (**a**) and its hydrolysate (**b**)

The protein and its hydrolysate produced under optimal conditions were analyzed using high-performance gel-filtration chromatography (Fig. 3). As anticipated, the protein of RBC was rich in peptide fractions with a molecular weight higher than 10 kDa (Fig. 3a), corresponding to the hemoglobin and globin. Whereas red blood cell hydrolysate (RBCH) was mainly composed of lower molecular weight peptides, with a noticeable amount of 94.79% lower than 10 kDa (Fig. 3b). Approximately 26.65% of the peptides in the hydrolysate had a molecular mass between 10 kDa and 3 kDa, and the fraction with a molecular mass lower than 3 kDa was 68.14%. The difference of molecular weight distributions between RBC and RBCH suggested that the enzymatic hydrolysis rendered mostly peptides smaller than 3 kDa. The obvious downward shift from macromolecular proteins to low molecular weight peptides, implied that the blood protein after hydrolysis could be of high nutritive value [24].

On the other hand, the uniqueness in distribution of peptide fractions might contribute to special functionalities and characterizations of the protein. It is well established that these peptides (< 6 kDa) may have the bioactive activities, such as antioxidative, antimicrobial, and antihypertensive functions [25, 26]. As shown in Fig. 3, BCH was abundant in peptide fractions and amino acid residues, implying its potential biological properties.

### Amino acid profile

The amino acid composition and chemical scores of RBCH are shown in Table 2. Overall, the amino acid profiles of RBC and RBCH were similar, while the former was slight lower than that of latter. The essential amino acids of RBCH accounted for more than 50% of the total amino acids, which was well above 36%, considering adequate for an ideal protein [27]. A generally accepted method to evaluate the nutritional quality of a protein is chemical score, which compares the levels of essential amino acids between test and standard proteins [28, 29]. We thus computed the chemical scores of RBCH based on the essential amino acids reference standards of FAO/WHO [30] for children (2–5 years old) and adults. In the case of children, the chemical scores of RBCH showed that isoleucine was the most limiting amino acids, and others (except methionine and threonine) were mainly present in sufficient or excess quantity for meeting their requirements. As for the adults, the essential amino acids except isoleucine could cover daily requirements. As the N Bhaskar and N Mahendrakar [28] demonstrated, the nutritive value of any high-quality protein depended on the capacity to fulfill the requirements of organisms. Therefore, in spite of minor deficiencies in certain essential amino acids, RBCH has the potential to be a nutritional ingredient used in the food and feed industry.

**Table 2:** Total amino acid compositions of red blood cell (RBC) and its hydrolysate (RBCH)

Item	RBC	RBCH	Chemical score of RBCH			
			RP-1^1^	RP-2^2^	RP-1	RP-2
Amino acids (g/100 g)						
Histidine	4.98	5.25	1.90	1.60	2.76	3.28
Isoleucine	0.32	0.34	2.80	1.30	0.12	0.26
Leucine	9.31	9.82	6.60	1.90	1.49	5.17
Lysine	6.00	6.31	5.80	1.60	1.09	3.94
Methionine	1.01	1.02	2.52^3^	1.70^3^	0.71	1.06
Phenylalanine	4.93	5.17	6.33^4^	1.90^4^	1.09	3.64
Threonine	2.74	2.83	3.40	0.90	0.83	3.15
Valine	5.84	6.10	3.50	1.30	1.74	4.69
Arginine	2.80	2.94				
Tyrosine	1.64	1.74				
Proline	2.30	2.47				
Alanine	5.58	5.87				
Aspartic acid	7.90	8.30				
Cystine	0.71	0.78				
Glutamic acid	6.32	6.62				
Glycine	3.12	3.29				
Serine	3.02	3.18				

^1^Essential amino acids for children (2–5 years old) according to FAO/WHO (1985)

^2^Essential amino acid for adults according to FAO/WHO (1985)

^3^Methionine + cystine

^4^Phenylalanine + tyrosine

### Functional properties of hydrolysate

#### Solubility

**Fig. 4:**
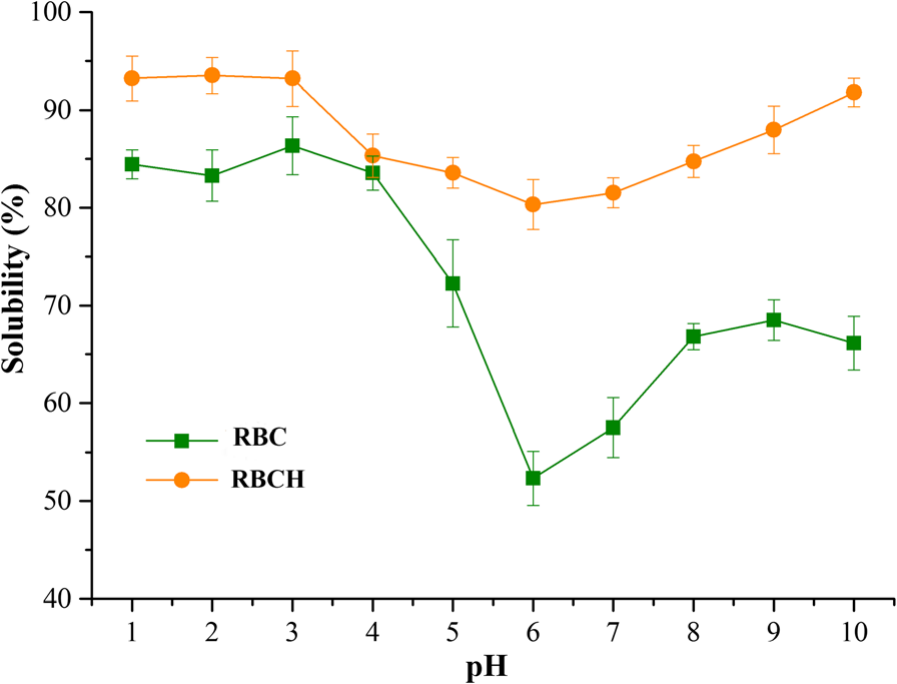
Solubility of red blood cell hydrolysate measured at different pH values

Fig. 4 shows protein solubility profiles of RBC and RBCH in the pH ranges of 1.0–10.0. Both showed similar solubility profiles that exhibited a V-shaped curve, revealing remarkable pH-dependent solubility. RBC and RBCH had the lowest solubility at pH 6.0 which might be ascribed to the isoelectric region, while the solubility increased on either side of this pH value. Compared with the native protein, the hydrolysate was soluble highly over a wide pH range with more than 80% solubility, corresponding to the findings of SY Naqash and R Nazeer [31], B Giménez, A Alemán, P Montero and M Gómez-Guillén [32] and M Chalamaiah, T Jyothirmayi, PV Diwan and BD Kumar [33]. This elevated solubility after hydrolysis was due to the degradation of proteins to low molecular weight peptides, which were expected to have proportionally more polar residues than the parent proteins, with the capacity of forming hydrogen bonds with water and thus increasing solubility [10, 11, 34].

#### Emulsifying properties

Emulsifying activity index (EAI) and emulsion stability index (ESI) of RBC and its hydrolysate at various pH are shown in Table 3. Approximately 33 m^2^/g of EAI at different pH ranges was observed in the case of RBC, which displayed no significant difference (*p* > 0.05) with increasing pH values. However, it was significantly lower than that of RBCH (*p* < 0.05) at all pH values except at pH 4 and pH 6 where the RBC exhibited higher emulsifying capacity. With regard to the hydrolysate, maximum EAI was 71.99 ± 1.18 m^2^/g at pH 10 and minimum was at pH 6 (13.93 ± 0.53 m^2^/g). Interestingly, despite RBCH having more smaller peptides than RBC (Fig. 3), it still had higher EAI values as the pH was further away the isoelectric regions. The behavior seems to contradict against view that the size and molecular weight of peptides play positive effect on the emulsifying properties [35, 36]. As HG Kristinsson and BA Rasco [37] reported, whereas in fact there was no explicit relationship between the peptide length and emulsification, the physicochemical characters of peptides might play an important role in their functional properties.

**Table 3:** Emulsifying activity index (EAI, m^2^/g) and emulsion stability index (ESI, min) of red blood cell (RBC) and its hydrolysate (RBCH) with different pH values ^1^

pH	EAI (m^2^/g)		ESI (min)	
	RBC	RBCH	RBC	RBCH
2	34.37 ± 2.56abB	51.24 ± 0.89bA	30.19 ± 2.44aB	80.47 ± 2.65aA
4	33.1 ± 1.18abA	24.12 ± 1.03cB	21.11 ± 0.69bcB	65.19 ± 2.81bA
6	32.18 ± 1.29bA	13.93 ± 0.53 dB	16.71 ± 0.58 dB	23.21 ± 1.78dA
8	35.45 ± 1.52aB	51.89 ± 0.63bA	22.33 ± 1.23bB	52.99 ± 0.33cA
10	33.66 ± 0.92abB	71.99 ± 1.18aA	19.34 ± 0.60cB	23.34 ± 1.52dA

^1^Average ± standard deviation (n = 3). Within columns, values with same lowercase letters are not significantly different at *p* > 0.05. Within rows, values with same uppercase letters are not significantly different at *p* > 0.05

Likewise, the lowest ESI of both RBC and RBCH were found at pH 6.0, with coincidental decrease in solubility. This phenomenon was mainly attributed to the net charge of the protein molecules or the decreased solubility around the isoelectric point [38]. RBCH exhibited much stronger emulsion stability than RBC at all observed pH values, which might be explained by the unique properties of hydrolysate and the mixed enzyme systems.

#### Foaming properties

Foaming capacity and foaming stability of protein and peptide hydrolysate were measured in different pH values (Table 4). Foaming capacity of RBC was significantly lower than that of RBCH (*p* < 0.05), possibly due to the higher solubility and flexibility of hydrolysate to form good foam by increasing the interaction at the air-water interface and reducing surface tension. Both had the lowest foaming capacity values at pH 6.0, corresponding to the emulsifying properties. The highest values observed at pH 2.0 were 12.67 ± 0.94% and 39.38 ± 0.88% for RBC and RBCH, respectively. Although we are not aware of comparable results for RBCH, the similar behaviors on foaming properties of hydrolysates from other by-products have been observed by researchers [31, 35]. Conversely, V Klompong, S Benjakul, D Kantachote and F Shahidi [10] found that foaming capacity of hydrolysate decreased at very acidic or alkaline pH due to the ionic repulsion of peptides. Those paradoxical results seem to be involved in the composition and net charge of peptides.

**Table 4:** Foaming capacity (%) and foaming stability (%) of red blood cell (RBC) and its hydrolysate (RBCH) with different pH values ^*a*^

pH	Foaming capacity (%)		Foaming stability (%)	
	RBC	RBCH	RBC	RBCH
2	12.67 ± 0.94aB	39.38 ± 0.88aA	9.83 ± 0.89aB	30.00 ± 1.41aA
4	11.50 ± 0.24bB	28.75 ± 0.35bA	9.66 ± 0.05aB	16.00 ± 1.71cA
6	3.33 ± 0.01eB	10.25 ± 0.35dA	0.67 ± 0.09cB	8.50 ± 0.38dA
8	6.67 ± 0.14 dB	26.50 ± 0.71cA	5.67 ± 0.94bB	18.75 ± 1.06bA
10	10.00 ± 0.94cB	28.75 ± 0.35bA	6.33 ± 0.01bB	19.50 ± 1.41bA

^a^Average ± standard deviation (*n* = 3). Within columns, values with same lowercase letters are not significantly different at *p* > 0.05. Within rows, values with same uppercase letters are not significantly different at *p* > 0.05

Foam expansions after whipping were monitored for 60 min to evaluate the foaming stability. Both RBC and its hydrolysate presented the comparable trends on the foaming stability with an increase of pH values. Further, there are notable differences in the values of foaming stability between RBC and RBCH. The latter exhibited a relatively high foaming stability, giving the credit to the thick cohesive layer around the air bubble formed by peptides. The lowest values were found at pH 6.0 for these two samples, which coincided with the precipitation of the large protein molecules at their isoelectric pH. Hence, solubility made a considerable contribution to the foaming behaviors as well as emulsifying properties.

### Antioxidant activity of hydrolysate

#### DPPH radical scavenging activity

**Fig. 5:**
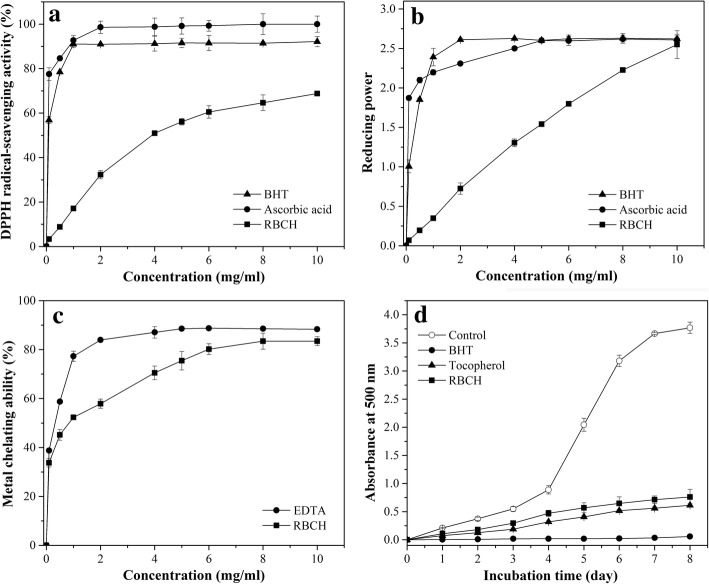
DPPH radical scavenging activity (**a**), reducing ability (**b**), metal chelating activity (**c**) and lipid peroxidation inhibition activity (**d**) of red blood cell hydrolysate (RBCH) at different concentrations

DPPH radical scavenging activities of hydrolysates at different concentrations (0–10.0 mg/mL) were measured and depicted in Fig. 5a. Clearly, the DPPH radical scavenging activity showed a concentration dependency, increased with increasing concentration and thereafter reached a plateau. This indicated that RBCH possessed hydrogen donating capability to suppress the radical chain reaction and convert DPPH radicals to harmless substances. The IC_50_ value of RBCH was 4.16 mg/mL, which was similar to those peptides like PYFNK (4.11 mg/mL) [39] and GVPLT (4.54 mg/mL) [40] derived from protein hydrolysates. Many researchers reported that low molecular weight peptides and some amino acids, with higher chance to cross the intestinal barrier and exert biological effects, possessed strong radical-scavenging activity [40–42]. Consequently, we presume that the small peptides and amino acids in hydrolysate may at least partially contribute to the DPPH radical scavenging activity.

#### Reducing power

Similarly, RBCH exhibited significant reducing ability with a dose-response relationship. As Fig. 5b showed, there was a linear and positive correlation between reducing power and concentration of RBCH. When the hydrolysate concentration increased from 0.1 to 10 mg/mL, the absorbance at 700 nm represented the reducing ability was an almost linear increase from 0.07 to 2.55. The reducing power obtained in this work was higher than that of muscle protein hydrolysate [31], rapeseed peptides [43] and cod protein hydrolysate [44] under the same concentration, but it was similar to the absorbance of 0.69 at 2 mg/mL for a hydrolysate derived from alfalfa leaf proteins [45]. Although RBCH displayed outstanding reducing ability, it was still inefficient compared to BHT and ascorbic acid. Within the concentration ranges of 0.50–10.0 mg/mL, the reducing power of RBCH was lower than those of BHT and ascorbic acid, and then got close to them at the highest tested concentration. Overall, the reducing power results revealed that RBCH possessed excellent reducing capacity to render hydrogen and electron which was involved in the antioxidant activity.

#### Metal chelating activity

Transition metals such as Fe^2+^ and Cu^2+^, as we all know, are inducers of lipid peroxidation and their chelation facilitates the antioxidation and subsequently prevents food rancidity [46]. We thus evaluated the metal chelating activity of RBCH against ferrous ion with EDTA as a positive control.

The ferrous ion-chelating effects of RBCH and EDTA are shown in Fig. 5c. RBCH had a strong chelating activity to capture ferrous ion to interfere with the formation of ferrous and ferrozine complex. The chelating activity of RBCH elevated rapidly at the beginning of concentrations (0–4 mg/mL), and afterwards increased slightly until a plateau was reached. It displayed approximately 75% chelating effect on Fe^2+^ ion at a concentration of 5 mg/mL, which was higher than that of hydrolysate derived from pacific hake muscle [47]. The reason may be explained by the possibility that the specific peptide structure and amino acid side chain groups play important roles in terminating the free radical chain reactions and chelating transition metal ions. Peptides derived from many protein sources with abundant hydrophobic amino acids were related with metal-chelating ability [48, 49]. The hydrophobic amino acids of RBCH accounted for more than 40% of the total amino acids, which might exert strong ferrous ion chelating ability. In addition, RBCH rich in histidine and histidine-containing peptides was presumed to have a significant effects on the chelating activity, in accordance with the report of L You, M Zhao, JM Regenstein and J Ren [50]. Our data implied that RBCH had an effective capacity for iron binding, suggesting that its action as an antioxidant might be associated with its iron binding ability.

#### Lipid peroxidation

Lipid peroxidation can cause deleterious effects in foods via forming secondary breakdown products of lipid peroxides and further generate rancid and toxic products. Therefore, the antioxidant activity of RBCH was measured in linoleic acid system and compared with those of tocopherol and BHT. As shown in Fig. 5d, the control had the highest absorbance value in this experiment, indicating the highest degree of oxidation among the samples after an 8-day incubation, whereas RBCH markedly retarded lipid peroxidation in the linoleic acid emulsion system. The strongest inhibition activity of linoleic acid peroxidation was about 80% which was too close to tocopherol, while it was much lower than that of synthetic antioxidant BHT. This result is similar to those of wheat germ protein hydrolysate [51] and BNH-P7 derived from blue mussel protein [20]. Though the synthetic antioxidants like BHT exhibit higher potent on inhibition of lipid peroxidation, they are restricted at higher concentrations for their toxicological factors [52]. In other words, RBCH with excellent antioxidant activity could be used at high concentration without side effects, and simultaneously impart desirable nutritional and functional properties to the final products. The strong inhibition potency of RBCH against linoleic acid might be attributed to enzymatic hydrolysis, which might give rise to both the release of antioxidant sequences and the exposition of previously hidden amino acid residues and side chains with antioxidant activity.

## Conclusion

The combination of neutrase and flavourzyme exhibited synergetic effects on the hydrolysis of protein derived from duck blood. The optimum conditions for degradation were temperature 51 °C, substrate concentration 14% (*w*/*v*), pH 7.0, and time 7.5 h. Hydrolysate prepared under the optimal conditions was characterized by having abundant small peptides (< 3 kDa, 68.14%) and high essential amino acid contents to fulfill human requirements. The spray-dried hydrolysate had enhanced solubility, higher emulsifying and foaming properties. RBCH also showed excellent DPPH radical-scavenging activity, reducing power and metal chelating activity to retard the lipid peroxidation. Therefore, a process for bioconversion of poultry waste was established, and the obtained peptide hydrolysate had the potential to be applied in food systems as a natural additive with good functional properties and antioxidant activity.

## Additional file

Additional file 1: **Table S1.** Analysis of variance for the regression model of DH. (DOCX 34 kb)

## Abbreviations

BHT: Butylated hydroxyltoluene
CCD: Central composite design
DH: Degree of hydrolysis
DPPH: 2,2-diphenyl− 1-picrylhydrazyl
EAI: Emulsifying activity index
ESI: Emulsion stability index
RBC: Red blood cell
RBCH: Red blood cell hydrolysate
ROS: Reactive oxygen species
RSM: Response surface methodology
