# Supplementary material for: Bioconversion of duck blood cell: process optimization of hydrolytic conditions and peptide hydrolysate characterization
Source: BMC Biotechnol. 2018 Oct 20;18:67. doi: 10.1186/s12896-018-0475-5 (PMC6196028; doi:10.1186/s12896-018-0475-5)
Supplement: Supplementary file 1 — Table S1. Analysis of variance for the regression model of DH. (DOCX 34 kb) [file 12896_2018_475_MOESM1_ESM.docx]

**Bioconversion of duck blood cell: process optimization of hydrolytic conditions and peptide hydrolysate characterization**

Zhaojun Zheng^a^, Xubiao Wei^a^, Tingting Shang^a^, Yan Huang^b^, Cong Hu^a^, Rijun Zhang^a^*

^^[[1]](#footnote-1)^a^ State Key Laboratory of Animal Nutrition, College of Animal Science and Technology, China Agricultural University, Beijing 100193, China

^b^ Department of Computer Science, College of Information and Electrical Engineering, China Agricultural University, Beijing 100083, China

^*^Corresponding author

Rijun Zhang, E-mail: feedbiotech@gmail.com

**Table S1.** Analysis of variance for the regression model of DH.

| Source | Sum of Squares | DF^a^ | Mean Square | *F* Ratio | Prob > *F* | Significant |
| --- | --- | --- | --- | --- | --- | --- |
| Model | 2352.995 | 13 | 181.000 | 469.47 | <.0001 | *** |
| Main (linear) effects |  |  |  |  |  |  |
| $x_{1}$ | 4.232 | 1 | 4.232 | 10.98 | 0.0011 | *** |
| $x_{2}$ | 414.222 | 1 | 414.222 | 1074.40 | <.0001 | *** |
| $x_{3}$ | 1.316 | 1 | 1.316 | 3.41 | 0.0659 | * |
| $x_{4}$ | 261.885 | 1 | 261.885 | 679.27 | <.0001 | *** |
| Interaction effects |  |  |  |  |  |  |
| $x_{1}$·$x_{2}$ | 8.406 | 1 | 8.406 | 21.80 | <.0001 | *** |
| $x_{1}$·$x_{3}$ | 2.445 | 1 | 2.445 | 6.34 | 0.0125 | ** |
| $x_{2}$·$x_{3}$ | 3.433 | 1 | 3.433 | 8.90 | 0.0031 | *** |
| $x_{1}$·$x_{4}$ | 4.172 | 1 | 4.172 | 10.82 | 0.0012 | *** |
| $x_{2}$·$x_{4}$ | 3.857 | 1 | 3.857 | 10.00 | 0.0018 | *** |
| Squared effects |  |  |  |  |  |  |
| $x_{1}^{2}$ | 10.073 | 1 | 10.073 | 26.13 | <.0001 | *** |
| $x_{2}^{2}$ | 1637.747 | 1 | 1637.747 | 4247.97 | <.0001 | *** |
| $x_{3}^{2}$ | 52.110 | 1 | 52.110 | 135.16 | <.0001 | *** |
| $x_{4}^{2}$ | 13.394 | 1 | 13.394 | 34.74 | <.0001 | *** |
| Lack of fit | 2.802 | 11 | 0.255 | 1.123 | 0.467 |  |
| Pure error | 1.362 | 6 | 0.227 | - | - |  |
| Residual (total error) | 4.164 | 17 | - | - | - |  |
| Correlation total | 2443.210 | 247 | - | - | - |  |

*R^2^* = 0.9631, Adjusted *R^2^* = 0.9610.

*, **, *** denote respectively significance level α = 0.1, α = 0.05, α = 0.01.

^a^ Degree of freedom.

1. [↑](#footnote-ref-1)
